# A Novel Variant in the Calcium-Sensing Receptor Associated with Familial Hypocalciuric Hypercalcemia and Low-to-Normal PTH

**DOI:** 10.1155/2020/8752610

**Published:** 2020-09-30

**Authors:** Sachin K. Majumdar, Tess Jacob, Allen Bale, Allison Bailey, Jeffrey Kwon, Terence Hughes, Andrea L. Barbieri, William Laskin, Paul Cohen, Tobias John Eric Carling

**Affiliations:** ^1^Department of Endocrinology, Bridgeport Hospital, Yale New Haven Health System, Bridgeport, CT, USA; ^2^Department of Internal Medicine, Bridgeport Hospital, Yale New Haven Health System, Bridgeport, CT, USA; ^3^Department of Pathology, Yale University School of Medicine, New Haven, CT, USA; ^4^Department of Radiology, Bridgeport Hospital, Yale New Haven Health System, Bridgeport, CT, USA; ^5^Section of Endocrine Surgery, Department of Surgery, Yale University School of Medicine, New Haven, CT, USA

## Abstract

Familial hypocalciuric hypercalcemia (FHH) is considered a relatively benign condition characterized by mild elevations in serum calcium and relatively low urinary calcium excretion. It results from an elevated set point in serum calcium arising from variants in the calcium-sensing receptor (CaSR) gene but also AP2S1 and GNA11 genes, which encode for adaptor-related protein complex 2 and G11 proteins, respectively. The manifestations of FHH can vary and sometimes overlap with primary hyperparathyroidism making the diagnosis challenging. *Case Presentations*. We report a mother and daughter with a novel heterozygous variant in the CaSR gene resulting in a serine to leucine substitution at position 147 (S147L) of the CaSR. Both patients had mild hypercalcemia, relatively low urinary calcium excretion, elevated calcitriol, and low-to-normal intact PTH. The proband (daughter) presented with symptoms associated with hypercalcemia and was incidentally found to have a bony lesion suspicious for osteitis fibrosa cystica, and she was also diagnosed with sarcoidosis. Subtotal parathyroidectomy revealed normal-weight parathyroid glands comprised of 50–80% parathyroid epithelial cells, which has been documented as within the spectrum of normal. Her mother had no symptoms, and no intervention was pursued. *Conclusion*. We report a novel variant in the CaSR associated with FHH in two patients with similar biochemical features yet differing clinical manifestations. While the relationship of the bony findings and parathyroid histology with this variant remains unclear, these cases enrich our knowledge of CaSR physiology and provide further examples of how varied the manifestations of FHH can be.

## 1. Introduction

The calcium-sensing receptor (CaSR) is present in all vertebrates where its principal function is to regulate serum calcium concentration by modulating parathyroid hormone and urinary calcium excretion [1]. A number of variants in the CaSR have been reported in humans associated with disorders of calcium balance, particularly familial hypocalciuric hypercalcemia (FHH), where the setpoint for serum calcium concentration is altered resulting in elevated serum calcium and relatively low renal calcium excretion [2]. Were it not for the inclusion of calcium in routine laboratory testing, most patients with mild primary hyperparathyroidism (PHPT) and FHH would not be identified given their largely asymptomatic nature, yet when mild hypercalcemia is detected, it is appropriate to exclude FHH, given that surgical management is rarely necessary. However, the phenotypes of FHH differ in their degree of calcium and PTH concentrations and even symptomatology, based on the particular variant in the CaSR, and can overlap clinically with PHPT [3–5]. Herein, we report two patients with FHH and a novel variant in the CaSR associated with low to normal PTH, elevated calcitriol, and symptoms associated with hypercalcemia that resolved after surgical parathyroidectomy in one patient.

## 2. Case Report

A 26-year-old woman of Korean and Irish descent was evaluated for chronic hypercalcemia and complaints of fatigue, body and joint aches, and dental pain for ∼4 years. She had no history of kidney stones or other medical problems except for multiple dental caries. Her mother had been treated for tuberculosis in Korea, and her maternal aunt had neck surgery in Korea at ∼age 70 reportedly for a parathyroid problem. Physical examination revealed a BMI of 23.3, BP 114/68, P 68, weight 63.6 kg, and absence of dental or jaw lesions and was otherwise unremarkable.

**Figure 1:**
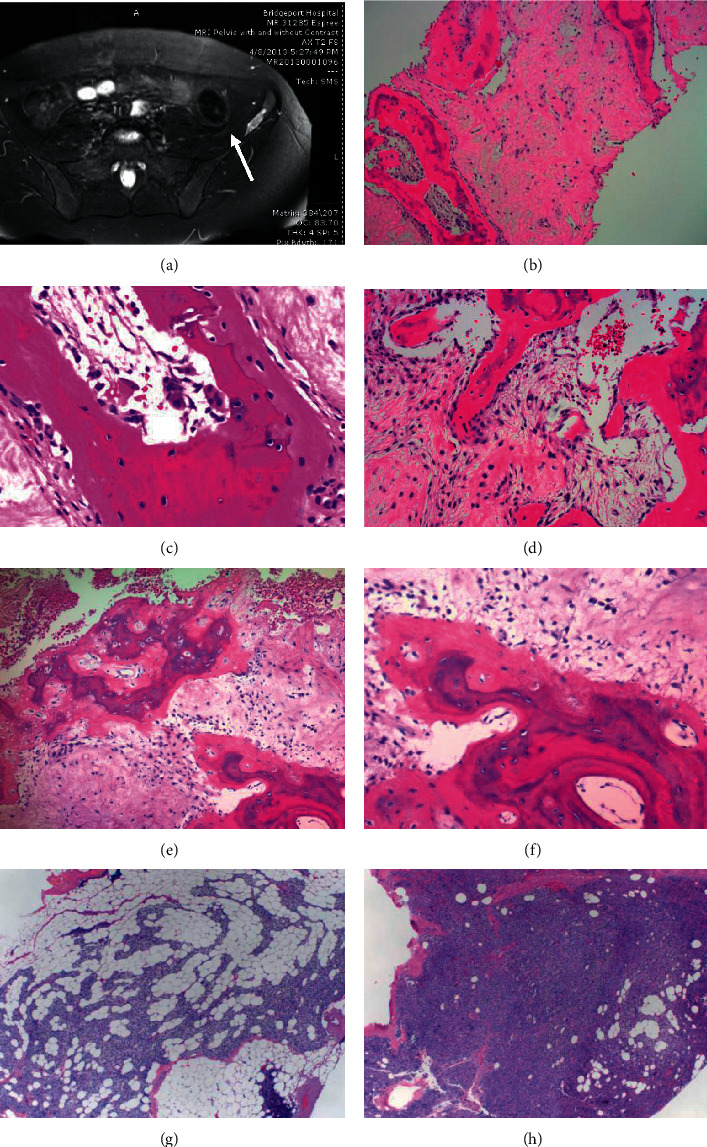
Bone imaging and bone and parathyroid histology. (a–f) Bone. (a) Initial MRI in 2013 showing bony lesion on the left iliac wing (bright area, white arrow) and (b–d) bone biopsy from 2013 reported as consistent with osteitis fibrosa cystica. Woven bone with marrow fibrosis (b), multinucleated osteoclasts with bone resorption (c), and osteoblastic rimming of new woven bone (d). (e) Repeat biopsy showing woven bone and peritrabecular fibrosis, with magnified portion (f). (g, h) Parathyroid. (g) Left lower parathyroid partial resection H&E stain, the specimen weighed 20 mg and measured 0.4 cm. Parenchyma was approximately 50% parathyroid epithelial cells (predominantly chief cells) and 50% adipocytes. (h) Right upper parathyroid gland biopsy, H&E stain, the specimen weighed 16 mg and measured 0.6 cm. Compared to that in (g), the parathyroid parenchyma is composed of approximately 80% parathyroid epithelial cells (predominantly chief cells) and 20% adipocytes. The amount of cellularity may be seen in normal parathyroid tissue or in hyperplasia. An additional biopsy of the left upper parathyroid gland was performed and was within normal limits based on weight and cellularity (not shown).

Laboratory studies 4 years prior to presentation revealed calcium levels of 10.3 mg/dL (8.4–10.2 mg/dL) and 10.8 mg/dL, phosphorus 2.4 mg/dL (2.5–4.5 mg/dL), 25 vit D 22 ng/dL (10 to 100 nM), albumin 4.4 g/L (3.4–5.4 g/dL), chloride 106 mM (96–106 mM), and creatinine 0.63 mg/dL (0.52–1.04 mg/dL). She underwent extensive evaluation over 2 years with results of blood and urine studies shown in Table 1 (daughter). Studies revealed mildly elevated calcium and calcitriol and low normal PTH. PPD and chest X-ray were negative, and urine testing favored FHH; however, she refused genetic testing citing the literature on the Internet, suggesting that PHPT is often misdiagnosed as FHH. CT imaging was performed to assess kidney stones, which were absent but, instead, showed a ∼3 cm indeterminate lesion of the left iliac wing. It appeared benign on MRI (Figure 1(a)), and subsequent biopsy revealed results consistent with osteitis fibrosis cystica (Figures 1(b)–1(d)). This prompted concern for an atypical case of PHPT with low PTH levels. Further testing revealed low normal values for PTH by dilution, mid-molecule PTH, and PTHrp (Table 1). Dual-energy X-Rray absorptiometry (DXA-Hologic) revealed bone densities and *Z* scores of 0.717 (*Z* − 1.2), 0.81 (Z − 2.1), 0.797 (Z − 1.2), and 0.346 (Z − 1.4), in g/cm^2^, at the femoral neck, spine, hip, and ultra distal forearm, respectively. Parathyroid Tc-99 sestamibi revealed equivocal tracer retention in the right thyroid lobe, yet neck ultrasonography revealed several lymph nodes but no parathyroid adenoma. A 4D CT scan of the neck showed mild prominence of the parathyroid glands and unexpected bilateral mediastinal and hilar lymphadenopathy.

Pulmonary lymph node biopsy revealed noncaseating granulomas consistent with sarcoidosis. A two-month trial of prednisone, 60 mg/d tapered to 20 mg/d, resulted in a normalization of calcitriol, 61 pg/mL and 37 pg/mL (18–72 pg/mL), at weeks 4 and 6, respectively, but no change in calcium, 10.9 mg/dL and 10.8 mg/dL, or PTH, 13 pg/mL and 16 pg/mL (10–65 pg/mL), respectively. Subsequent CT scanning of the chest revealed resolution of lymphadenopathy, and given the combination of elevated calcium, normal PTH, and low urine calcium, suspicion for FHH remained. The bone pathology was reviewed extensively and remained curious, yet its interpretation was not strictly limited to osteitis fibrosis cystica. At this time, she refused further medical evaluation and sought consultation from an endocrine surgeon.

The case was presented and discussed at a multidisciplinary treatment conference, and the patient was counselled by the endocrine surgeon that parathyroid surgery was unproven in this scenario but could be considered based on her symptoms and she requested to proceed with parathyroidectomy [6]. She underwent a subtotal parathyroidectomy with intraoperative PTH measurements, with a drop from 32 pg/mL to 9 pg/mL, 10 minutes after subtotal resection, leaving a remnant of approximately 20 mg of the left lower parathyroid gland. Intraoperatively, the parathyroid glands appeared minimally hyperplastic, and upon histologic review, they were each composed of 50–80% parathyroid epithelial cells with the rest composed of adipose tissue. Final pathology demonstrated that the left and right upper and left lower parathyroid glands weighed 20, 16, and 15 mg, respectively; two appeared histologically normal and one was hypercellular (Figures 1(g) and 1(h)). She developed postoperative hypoparathyroidism, with calcium 7.2 to 9.1 mg/dL and PTH < 3 to 5 pg/mL, but was maintained on calcitriol and reported an overall improvement in wellbeing and resolution of fatigue and body aches.

The patient referred her mother since she was noted to have hypercalcemia based on further inquiry. Her mother, a 62-year-old Korean woman with a history of treated tuberculosis, hypertension, and hyperlipidemia had mild asymptomatic hypercalcemia. Her calcium levels from 1997 to 2014 ranged from 10.2 mg/dL to 11.3 mg/dL, and further testing revealed similar findings to her daughter (Table 1, mother). CT imaging of the chest and abdomen did not reveal kidney stones or evidence of sarcoidosis, and the neck US revealed a thyroid nodule found to be benign on FNA. Surgery was discouraged, and she agreed to genetic testing revealing a novel heterozygous variant in the calcium-sensing receptor gene (CaSR) resulting in a serine to leucine substitution at position 147 (S147L). After this, her daughter agreed to testing which revealed the same heterozygous variant in the CaSR.

On follow-up, the probands bony lesion was re-biopsied, 3 years after the initial biopsy and 1.5 years after surgery, revealing woven bone deposition and peritrabecular fibrosis, and it has been stable on imaging over 5.5 years. Repeat DXA (Hologic) 5.5 years after her initial one, and 3.5 years after parathyroid surgery, revealed increased bone density and *Z* scores of 0.798 (*Z* − 0.3), 0.927 (*Z* − 1.1), and 0.870 (*Z* − 0.5) in g/cm^2^ at the femoral neck, spine and hip, respectively, although the study was performed at a different location. Three years postoperatively, she continues to report a resolution in symptoms since her surgery, particularly in fatigue and mental clarity, yet remains hypoparathyroid (calcium 7.8 mg/dL; PTH 4 pg/mL) and on treatment with calcitriol and calcium. She had an uncomplicated pregnancy resulting in a healthy male child whose calcium so far has not been tested. Her mother remains asymptomatic.

## 3. Discussion

We report a novel variant in the CASR gene (TCA > TTA: hg19chr2:121976182, NM_000388) resulting in a serine to leucine substitution at position 147 (S147L) associated with FHH and low to normal PTH. The initial evaluation of the proband was complicated by the presence of sarcoidosis, a bony lesion suspicious for osteitis fibrosa cystica, as well as symptoms of fatigue and bony pains. Interestingly, she reported resolution of her symptoms and improvement in mental clarity after subtotal parathyroidectomy and was satisfied with surgery despite being maintained on calcium and calcitriol. However, her mother was asymptomatic despite sharing similar biochemical findings.

Studies identify Serine 147 as an important site for calcium sensing; for example, in one structural study, a serine to alanine (S147A) substitution resulted in a 4-fold decrease in maximal response to calcium in vitro [7, 8]. The general findings in these two patients fit well within the spectrum of previously reported FHH phenotypes where other variants are associated with low to normal PTH concentrations and elevated calcitriol [9, 10]. Examination of the probands parathyroid tissue revealed 50–80% parathyroid epithelial cells in each gland. The significance of these findings is unclear, as autopsy studies have documented large variations in intraparathyroidal adipose tissue in normal patients at autopsy [11]. Sarcoid may have contributed to her elevations in calcitriol, yet in the mother, calcitriol was elevated without evident sarcoid and therefore may be a feature of this variant.

The proband's unexpected bone lesion in the iliac wing revealed pathology suggestive of osteitis fibrosis cystica and complicated the evaluation. While the CaSR is present in cartilage and bone and contributes to skeletal homeostasis, the bone pathology reported in rare variants of FHH is associated with excess PTH rather than being independent of it [12, 13]. This lesion was stable over 5 years on imaging, and repeat biopsy results were less specific suggesting that the finding may be unrelated yet parathyroidectomy may have altered its characteristics. Symptoms associated with hypercalcemia in the proband appeared real given their resolution after parathyroidectomy and absence after several years of follow-up. This is consistent with prior reports describing overlapping symptoms in FHH and PHPT. In an early description of 15 kindreds with FHH, affected members reported significantly more frequent fatigue, weakness, headaches, and arthralgia, in comparison to those unaffected [3, 14]. Given the absence of symptoms in the mother, we cannot argue that this particular variant is associated with symptom development, rather it may be a consequence of the inherent differences individuals possess in susceptibility to hypercalcemia-associated symptoms, perhaps independently of the cause.

In summary, these cases support experimental evidence that serine 147 is an important site on the CaSR for calcium sensing and that the S147L substitution is associated with clinical and biochemical features of FHH. This adds to the database of known variants in the CaSR providing further insight into genotype phenotype relationships in humans and supports the notion that FHH is quite variable biochemically and clinically, and can overlap with PHPT. Also, given that symptoms might occur in individuals with mild hypercalcemia and FHH, attempts to normalize the serum calcium either medically or surgically may occasionally be indicated ..

## Figures and Tables

**Table 1:** Range of values displayed when numerous results are available.

Measure	Reference	Daughter	Mother
Calcium	8.4–10.2 mg/dL	10.1.–11.1	10.2–11.3
Ionized calcium	4.4–5.5 mg/dL	5.7	5.8–6.0
Creatinine	0.52–1.04 mg/dL	0.54–0.69	0.5–0.61
Chloride/phosphate ratio		33–41	35
Phosphorus	2.5–4.5 mg/dL	2.1–3.4	3.1–3.3
Magnesium	1.6–2.3 mg/dL	2.2–2.4	2.2
Intact PTH	10–65 pg/mL	13–24	12–42
25-OH vit D	Variable ng/dL	20–22	25–42
1,25-OH vit D	18–72 pg/mL	72–94	67–81
24 hour urine calcium (grams creatinine/24 h)		103 (1.27 g Cr),	84 (1 g Cr)
		105 (1.65 g Cr)	90 (1.02 g Cr)
Ca/Cr clearance ratio		0.004	0.004
PTH by dilution		24, 24 (1 : 2), 27 (1 : 4)	
Midmolecule PTH	10–25 nLmeq/mL	11	10
ACE	9–67 U/L	34	38
Alk phos-bone specific	4.7–17.8 mcg/L	8.9	
CTX	64–640 pg/mL	239	

ACE, angiotensin converting enzyme; PPD, purified protein derivative; PTH, parathyroid hormone; CTX, collagen type 1 C-telopeptide.

## Data Availability

Data were obtained directly from the patients and their medical records.

## Additional Points

*Summary.* The clinical and biochemical features of FHH are described in a mother and daughter harboring a novel variant in the CaSR. The daughter (proband) had symptomatic hypercalcemia reversed by surgery and an unusual bony lesion, while her mother was asymptomatic and required no intervention. These cases illustrate the clinical variability of FHH and its occasional overlap with PHTP.
